# Supplementary material for: A Pilot Study on Ad Libitum Mediterranean Diet Intervention for Women with PCOS: A Mixed-Methods Exploration of Acceptability, Adherence, and Participant Lived Experience
Source: Nutrients. 2025 Mar 21;17(7):1105. doi: 10.3390/nu17071105 (PMC11990588; doi:10.3390/nu17071105)
Supplement: Supplementary file 1 [file nutrients-17-01105-s001.zip › Supplementary Material Table S1.pdf]

Supplementary Table S1. Survey and interview schedule developed to assess participants acceptability and lived experience of a MedDiet intervention.

	Very High	High	Average/Neutral	Low	Very low/none
How would you rate your knowledge of what foods are part of a Mediterranean diet?	☐	☐	☐	☐	☐
How would you rate your confidence to prepare/cook the food included in a Mediterranean diet?	☐	☐	☐	☐	☐
How would you rate your confidence toward having the time to cook/eat a Mediterranean diet?	☐	☐	☐	☐	☐
How would you rate your ability to afford foods that are required for a Mediterranean diet?	☐	☐	☐	☐	☐
How would you rate your access to the foods that are required for a Mediterranean diet?	☐	☐	☐	☐	☐
How would you rate the acceptability of a Mediterranean diet by your friends or family?	☐	☐	☐	☐	☐
How would you rate your ability to adhere to a Mediterranean diet?	☐	☐	☐	☐	☐
How would you rate your intention to follow a Mediterranean diet?	☐	☐	☐	☐	☐

**Resources/delivery**	Strongly agree	Agree	Neither agree nor disagree	Disagree	Strongly disagree
I found the education resources **easy** to understand	☐	☐	☐	☐	☐
I found the education resources **easy** to read	☐	☐	☐	☐	☐
I found the education resources **were** useful	☐	☐	☐	☐	☐
The education resources made it **easier** to adhere to a Mediterranean diet	☐	☐	☐	☐	☐
I would have liked **more** resources	☐	☐	☐	☐	☐
I found the dietary consultations **were** useful	☐	☐	☐	☐	☐
I found the text messages were helpful?	☐	☐	☐	☐	☐
I would have liked **more** text messages?	☐	☐	☐	☐	☐
The text messages were too **short**?	☐	☐	☐	☐	☐

Semi-structured interview questions

1. Could you describe to me what you think of as a Mediterranean diet?
   Prompt: which foods/beverages do you think are included?

2. What factors or circumstances would help you to follow a Mediterranean Diet?

3. What factors or circumstances would make it harder for you to follow a Mediterranean diet?

4. What skills do you think you will need to follow this diet?

5. In what ways do you think following a Mediterranean diet could affect your health/lifestyle?

6. How do you feel about following a Med Diet?
   Prompt: is it something that you enjoy or not? Do you look forward to it or not?

7. What was your view on the education resources?

8. Do you have any suggested improvements on the delivery of the dietary intervention and/or education resources/materials?

9. How did you feel about following a dietary approach independent of calorie intake or weight loss? *Prompt: did it make it easier or harder to adhere? Did you feel it would be helpful or not helpful for PCOS?*

10. Any final comments that were not described in previous questions?
