# Supplementary material for: A Pilot Study on Ad Libitum Mediterranean Diet Intervention for Women with PCOS: A Mixed-Methods Exploration of Acceptability, Adherence, and Participant Lived Experience
Source: Nutrients. 2025 Mar 21;17(7):1105. doi: 10.3390/nu17071105 (PMC11990588; doi:10.3390/nu17071105)
Supplement: Supplementary file 1 [file nutrients-17-01105-s001.zip › Supplementary Material Table S2.pdf]

Supplementary Table S2. Strategies for Mediterranean diet implementation aligned with the Behaviour Change Wheel [40]

COM-B component	Intervention function	Policy categories through which the intervention strategy will be delivered	Recommended intervention strategy and corresponding behaviour change technique number
Psychological capability	Education<br>Training<br>Enablement	Communication/marketing<br>Service provision	Dietitian led **education** and **training** on the MedDiet for PCOS to include:<br>o What constitutes a MedDiet (4.1)<br>o Debunk common myths about diet and PCOS (9.1)<br>o The role of a MedDiet for PCOS symptom management beyond weight loss (5.1)<br>o Strategies required to implement dietary changes including, developing personalised meal plans, setting incremental goals to achieve the recommended targets for food group quantities and servings and identifying suitable food substitutions (4.1, 1.4, 1.1, 8.2, 8.7)<br><br>Provision of practical resources that can be used in both hard copy and digital form, to support **training** and **enablement** of dietary implementation including:<br>o A pamphlet of health consequences/benefits for PCOS (5.1)<br>o Fact sheets of key MedDiet principals and cultural translation (6.1)<br>o Infographics of the serving size/quantities of recommended foods (4.1, 7.1)<br>o Recipe booklets (4.1, 6.1)<br>o Meal planning guides and templates (4.1)<br>o Shopping guides with food substitutions for cultural, taste and dietary preferences (7.1, 6.1)<br>o Fridge magnet of key principles as a visual prompt (12.5, 7.1)<br>o Weekly checklists for monitoring dietary adherence (2.3)<br><br>Provision of weekly one-way text messages that will **enable** dietary adherence by providing supportive and informative health prompts (7.1) *Content of messages* (4.1, 5.1, 3.1)<br><br>Provision of online **training** packages and workshops for Dietitians that include<br>o A summary of the evidence for a MedDiet in PCOS management (9.1, 5.1)

			o The benefits and strategies for prioritising diet quality over weight loss in PCOS management (4.1, 5.1)<br>o Tools and instructions for scoring MedDiet adherence (4.1, 6.1)<br>o Knowledge of patient-centred implementation strategies related to culture translation, patient capabilities, geographic location (e.g., rural) and socio-economic status (4.1, 6.1)<br>o Simple, visually appealing patient handouts to complement dietary consultation such as those listed above in the practical resource recommendation (9.1, 12.5)
Physical capability	Training	Service provision<br>Communication/marketing	Provision of engaging media (e.g. videos) to deliver interactive **training** workshops or cooking demonstrations to reinforce hands-on learning (4.1, 6.1)
Physical opportunity	Enablement<br>Education<br>Training<br>Persuasion<br>Incentivisation	Service provision<br>Guidelines<br>Communication/marketing	**Enable** dietary adherence by providing **education** and **training** to overcome practical barriers including:<br>o **Educate** individuals on budget-friendly and time-efficient MedDiet alternatives by promoting practical food swaps, such as using frozen or pre-cut vegetables, canned seafood, and pre-cooked grains to reduce preparation time and cost while maintaining nutritional value (1.2, 4.1)<br>o Provide guidance on cost-saving strategies, including buying staple ingredients in bulk, choosing plant-based protein sources, and prioritising seasonal produce to maximise affordability (1.2, 4.1)<br>o Develop and distribute budget conscious MedDiet meal plans with detailed cost comparisons to non-Mediterranean dietary patterns, highlighting practical ways to reduce expenses while following the diet (1.2, 4.1)<br>o Provide guidance on where to buy MedDiet foods to maximise quality and affordability such as farmers markets, local producers or value supermarkets (4.1)<br>o Provide a guide on selecting MedDiet meals when going out (4.1)<br><br>**Persuade** healthcare professionals to prioritise patient referrals to nutrition professionals at diagnosis and yearly by developing and distributing online presentations, fact sheets, and professional development workshops. These should demonstrate how nutrition support helps patients make sustainable dietary changes, manage their condition, build self-efficacy, and reduce short- and long-term health risks. Additionally, highlight government provided financial **incentives** available to physicians (9.1, 5.1, 5.6, 10.1)

			**Enable** patients **to self-educate** by directing them to evidence-based resources endorsed by credible health organisations e.g., AskPCOS application (9.1)
Social opportunity	Environment<br>Education<br>Training<br>Modelling	Communication/marketing<br>Service provision	Connect patients with PCOS support groups to improve the **environment** by offering practical and emotional support (3.1, 4.1)<br><br>Provide **education** and **training** on practical strategies for shared meals, such as bringing MedDiet-friendly dishes to social gatherings (4.1)<br><br>Encourage patients to find a healthy lifestyle buddy for reciprocal **modelling** of healthy lifestyle behaviours (3.1, 6.1, 13.1)<br><br>Change the **environment** by encouraging the sharing of meals and cooking experiences to facilitate conviviality and **model** healthy dietary behaviour (13.1, 12.2)<br><br>Provide **education** and **training** on communication strategies when encountering social pressure to eat foods which are unconducive to goals (e.g., via demonstration and role play) (6.1, 8.1, 4.1)
Automatic motivation	Education<br>Persuasion		**Persuade** patients through **education** to reframe the diet as a lifestyle that is not only health-enhancing but also fun, adventurous, and flexible. Emphasise enjoyment through variety and flavour (13.2)<br><br>**Persuade** patients to reframe healthy lifestyle behaviours as self-care strategies by reflecting on positive change such as increased energy, improved relationship with food, improved mood and better sleep (13.2)<br><br>**Persuade** patients through **education** that a MedDiet is a strategy that focuses on healthy behaviours independent of weight loss, unlike many diets recommended to patients who have PCOS and overweight (13.2)<br><br>**Educate** patients on mindful eating strategies to focus on the pleasure of eating rather than rules or restrictions (11.2, 13.2)

			**Educate** patients on the emotional consequences dietary choices by using a mood-based journal to visually reinforce the connection between dietary choices and emotions and feelings (5.4)<br><br>Direct patients to MedDiet friendly content creators who emphasize joyful, weight-neutral nutrition to **persuade** patients to reframe healthy eating as a positive experience (13.2)
Reflective motivation	Education<br>Enablement<br>Persuasion	Guidelines<br>Service provision<br>Communication/marketing	**Educate** health professionals to provide weight-neutral approaches for PCOS management and focus on adding healthy foods and habits rather than emphasising dietary restrictions (5.1, 5.6)<br><br>**Enable** the development of self-efficacy using goal setting to develop progressive and meaningful dietary behaviour change (1.1, 1.4, 8.7)<br><br>Provide **education** on health benefits beyond PCOS symptoms including mental wellbeing, quality of life (5.6)<br><br>Use motivational interview techniques to **enable** patients to identify the advantages or disadvantages of changing dietary behaviour and to reflect and focus on times they have successfully changed behaviour (9.2, 9.3, 15.3)<br><br>Use imagery of long-term health outcomes and goals to **enable** patients to maintain dietary adherence (9.3)

Behaviour change techniques and corresponding numbers according to the behaviour change taxonomy [49]: 1.1 Goal setting (behaviour) 1.2 Problem solving 1.3 Goal setting 1.4 Action planning 2.3 Self-monitoring of behaviour 3.1 Social support (unspecified) 4.1 Instructions on how to perform a behaviour 5.1 Information about health consequences 5.4 Monitoring of emotional consequences 5.6 Information about emotional consequences 6.1 Demonstration of the behaviour 7.1 Prompts/cue 8.2 Behaviour substitution 8.7 Graded tasks 9.1 Credible sources 9.2 Pros and Cons 9.3 Comparative imagining of future outcomes 10.1 Material incentive 11.2 Reduce negative emotion 12.5 Adding objects to the environment 13.1 Identification of self as role model
Abbreviations: COM-B, Capability, Opportunity, Motivation – Behaviour model
